# Win–win interactions: Results and implications of a user needs assessment of clinical and translational scientists

**DOI:** 10.1017/cts.2023.6

**Published:** 2023-02-07

**Authors:** Shannon Casey, Amanda Siebert-Evenstone, Allan R. Brasier

**Affiliations:** 1 Institute for Clinical and Translational Research, School of Medicine and Public Health, University of Wisconsin-Madison, Madison, WI, USA; 2 Age of Learning, Inc., USA; 3 Department of Medicine, University of Wisconsin–Madison, School of Medicine and Public Health, Madison, WI, USA

**Keywords:** Education, clinical research training, science of team science, qualitative methods

## Abstract

**Introduction:**

This study describes a needs assessment of clinical and translational research (CTR) scientists at a large, distributed, School of Medicine within a public university and affiliated clinics.

**Method:**

We performed an Exploratory Conversion Mixed-Methods analysis using a quantitative survey and qualitative interviews with CTR scientists across the training continuum, from early-career scholars, mid-career mentors, and senior administrators at the University of Wisconsin and Marshfield Clinics. Qualitative findings were confirmed using epistemic network analysis (ENA). A survey was distributed to CTR scientists in training.

**Results:**

Analyses supported that early-career and senior-career scientists have unique needs. Scientists who identified as non-White or female reported needs that differed from White male scientists. Scientists expressed the needs for educational training in CTR, for institutional support of career development, and trainings for building stronger relationships with community stakeholders. The tension between meeting tenure clocks and building deep community connections was particularly meaningful for scholars who identified as under-represented, including based on race, gender, and discipline.

**Conclusions:**

This study yielded clear differences in support needs between scientists based upon their years in research and diversity of identities. The validation of qualitative findings, through quantification with ENA, enables robust identification of unique needs of CTR investigators. It is critically important to the future of CTR that scientists are provided with supports throughout the career. Delivery of that support in efficient and timely ways improves scientific outcomes. Advocacy at the level of the institution for under-represented scientists is of utmost importance.

## Introduction

This report summarizes an Exploratory Conversion Mixed Methods [1] Needs Assessment (NA) conducted at the University of Wisconsin-Madison Institute for Clinical and Translational Research (ICTR) to identify the needs of clinical and translational research (CTR) scientists. Tremendous institutional and federal resources go into training future generations of CTR scientists in team science collaborations via the Clinical and Translational Science Awards Program [2]. To successfully attract and retain CTR scientists, hubs must provide meaningful research opportunities that (1) support career progression, (2) foster and promote team science, and (3) minimize institutional barriers that stall CTR [3]. The need for quick scientific turnaround was highlighted during the COVID-19 pandemic [4,5], which called for rapid movement of vaccine discovery and disease prevention into diverse communities. Because translational science requires intensive collaboration and can be protracted, it can be difficult for CTR scientists to meet traditional academic tenure clocks [6]. Our goal is to share our learning of scientist needs for training, research services, community building, and scientific outcomes with other CTSA hubs. Identifying the developing needs of scientists is central to understanding the future of translational clinician-scientist workforce development.

**Fig. 1.**
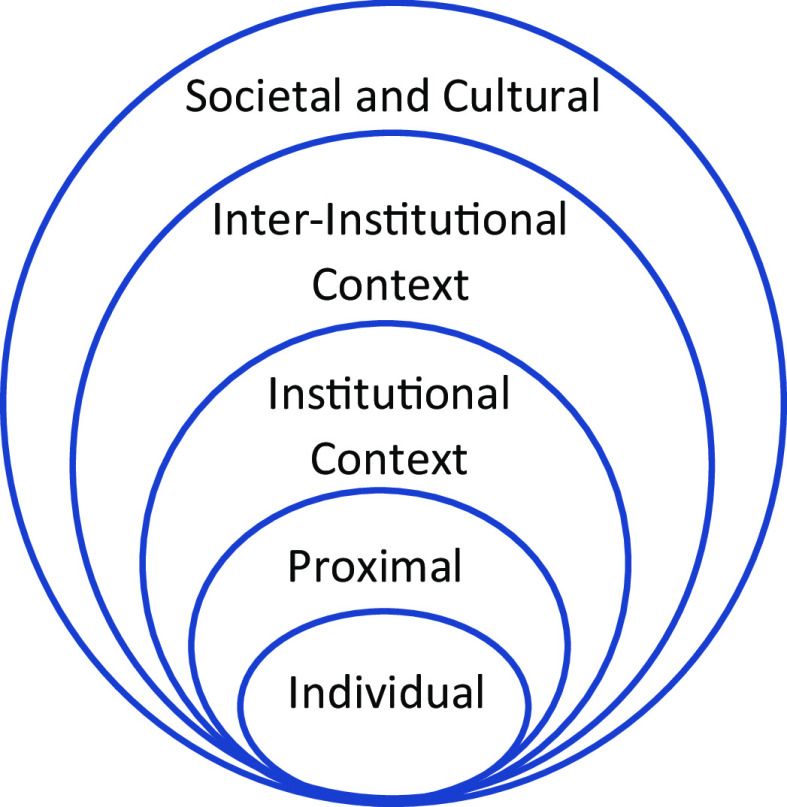
Ecological model of development. Bronfenbrenner, U. *The ecology of human development: Experiments by Nature and Design*. Harvard University Press, 1979.

In 2019, van Dijk and colleagues [6] summarized the challenges associated with building an academic career in medicine, including difficulties related to training, career evaluation, funding, and infrastructure. Effective CTR necessitates coordination and communication across multiple individuals, disciplines, settings, and institutions [7]. To create strong teams, scientists must be mentored by skilled team scientists and reach out to colleagues in other disciplines to pursue and realize grants [8]. It can be helpful to conceptualize individual scientist career development using Bronfenbrenner’s ecological model (Fig. 1) [9]. The ecological framework applied to CTR scientists illustrates the multiple layers of scientist development, (1) as individuals with unique identities and perspectives, (2) within influential interpersonal relationships such as with mentors, (3) operating in universities, institutions, and clinics with policies and rules, (4) at the service of individuals and communities who benefit from CTR, and (5) within broad social contexts including historical trends. Though all medical research can be viewed using an ecological lens, the complex nature of CTR requires deep forethought of each of those layers. Translational science teams are most successful when individuals collaborate across all of the ecological layers, with scientists from other disciplines, with individuals in other settings, and with individuals in diverse communities [10,11]. To train the next wave of CTR scientists, it is imperative that CTSA programs understand the specific, and specialized, needs of this highly educated group of innovators.

Multiple CTSAs have conducted needs assessments using surveys to identify training in research and CTR competencies [12,13]. Those studies summarize capacity-building needs in minority medical and health science institutions in Puerto Rico [14], Hawaii [13], and Rhode Island [12]. Findings support specific areas of training needs, including special needs based on the level of the respondent [14]. Recommendations from those studies include workshops and trainings across a broad range of topics, increased access to local data sources, data analysis training, pilot funding and grant support, and study design and statistical support [12]. Each study supported a different set of needs based on the scientist’s discipline, career-level, and setting (for example, large versus small institutions).

Between November 2020 and February 2021, our CTSA at the University of Wisconsin-Madison sought to understand, with an eye toward complexity, the needs of CTR scientists across our distributed network within the context of COVID-19. Our goal was to develop our training programs and services in close collaboration with, and ample input from, existing experts (defined as a current junior or senior translational scientist). To that end, we conducted a needs assessment with CTR researchers, aimed at: (1) understanding the state of support for CTR scientists at the UW-Madison and (2) establishing foundational evidence for ICTR plans for scientist training and services. A four-phase Exploratory Conversion Mixed-Methods Needs Assessment was designed and executed, with the goal of a more nuanced understanding of how individual needs and identities interact within diverse contexts, including those highlighted in the ecological model, to advance careers and scientific outcomes. Results of the study were intended to support our planning in advance of training grant renewals.

## Materials and Methods

**Fig. 2.**
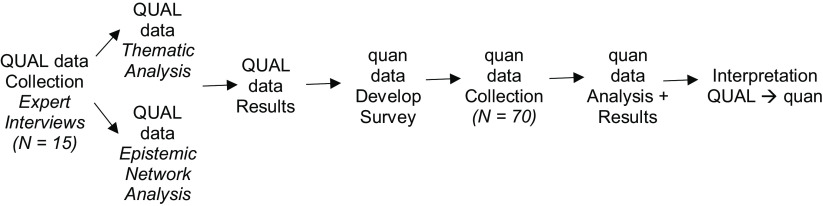
Exploratory conversion mixed analysis design. QUAL = Qualitative Research; Quan = Quantitative Research. Model based on Chapter 4: Choosing a mixed methods design from *designing and conducting mixed methods research* [52].

We launched the needs assessment by forming an Oversight Committee of five ICTR senior trainers, faculty, administrators, and evaluators. Phases of the needs assessment included: (1) a design phase to set the study purpose, primary research questions, and protocol; (2) an environmental scan to identify and review CTR knowledge from prior needs assessments, (3) a research phase of exploratory qualitative interviews with CTR experts followed by a brief quantitative survey of current ICTR trainees, and (4) interpretation and recommendations from CTSA experts for improving existing offerings and services. During the design phase, the Oversight Committee advised on the multilayered assessment and identified core research questions. Those questions centered on CTR scientist needs, gaps in training, and barriers to successful CTR. The environmental scan was conducted by members of the CTSA Evaluation and Tracking team. Multiple components (e.g., the Community Academic Partnerships, Dissemination & Implementation component [15], and the local National Research Mentoring Network) provided materials for review. After completion of the environmental scan, Oversight Committee members elected a mixed-method design (Fig. 2) [16], settling on a *Conversion Mixed Analysis.* 1 Conversion Mixed Analysis is an exploratory approach that uses qualitative and quantitative data, requiring analysis of either type of data in at least two ways. This study analyzed qualitative data using thematic analysis and statistical modeling of themes using ENA. That approach allowed for integration of multiple perspectives [17]: first qualitatively from experts with in-depth and historical familiarity with CTR and second quantitatively from current trainees. Careful planning and execution at all levels were critical for guaranteeing data quality and interpretation. Efforts were made across phases to elicit critical and constructive feedback to identify areas for intervention and innovation. Institutional Review Board members identified this study as data gathered for program development and not as protected human subjects research.

### Qualitative Interviews

The Oversight Committee created and tested the qualitative interview protocol with two senior CTR researchers. Qualitative interview questions were developed to elicit needs, gaps, and barriers related to the conduct of CTR. Questions included (1) a framing question *(Please describe ways you currently work with, advise, or observe (CTR) scientists in their work),* (2) a question related to needs and gaps *(What are pressing needs and challenges you see for CTR scientists navigating the lifecycle of CTR?)*, and (3) a question related to a CTSA’s ability to meet needs and close gaps *(What solutions do you see for meeting needs of CTR scientists; what could ICTR do to better support CTR scientists*?*)*. We used semi-structured interviews to engage flexibly with interviewees, following their prompting of key priorities and probing for additional detail. Interviews were conducted by a trained qualitative interviewer who has conducted mixed-method evaluations at numerous universities and public health departments.

Of 18 invited scholars, 15 participated in recorded, 30- to 45-minute one-on-one interviews, including Deans and Directors, faculty, and trainees from five schools (Medicine, Nursing, Education, Pharmacy, and Engineering) and two divisions (Health System and Clinical Research Institute). Interview participants ranged in age from early 30s to early 70s, were 53% female-identified, and included Academic Deans, School Deans, tenured Full and Associate Professors, tenure-track Assistant Professors, Research Fellows, post-doctoral graduate fellows, and Clinical Research professionals, with selection based upon prior experience engaging in the conduct of CTR. Interviewing people at all stages of career development allowed for triangulation of concepts from the perspective of administrative directors, tenure review committee members and chairs, as well as from those going through the process of building careers in research. Because the goal was expert perspectives, purposive sampling was used to maximize responses from our network of influential and experienced individuals [18]. We pursued interviewees until we heard repeat responses to our four semi-structured research questions [19].

Our goal was to reach thematic saturation with interviews [20,21], to ensure that critical needs had been identified. Experts in methodology express the importance of theoretical saturation as a criterion for judging the number of qualitative interviews required to explore health science [20]. Saturation is reached when no new or additional information is observed. Studies support that saturation can be reached in as little as 5, or as many as 15, interviews for phenomenological studies, with some finding data saturation occurs within 12 interviews [20]. Studies have identified that when informants are experts with an achieved level of competence [22], as defines our sample, a small number of informants (e.g., *n* = 7) is sufficient to correctly classify data. We delayed analytic foreclosure [23] by holding off on doing thematic coding until multiple interviews had been read multiple times.

The virtual interview platform allowed for verbatim transcripts of qualitative interviews, verified by interviewers watching, correcting, and confirming accuracy. Two independent coders did preliminarily reflexive thematic analysis [23,24], to develop themes that were conceptualized as patterns of shared meaning that highlighted CTR scientist needs (Table 1). Differences in coding were resolved by discussion between coders to ensure consistency. Interview transcripts were redacted for irrelevant content (e.g., a parent working remotely from home whose child asked for assistance captured on audio).

**Table 1.** Reflexive Thematic Analysis outcomes organized by systems level, thematic description, and interviewee career stage

Level	Theme	Interviewee career stage		Representative quotes
		Early-career	Senior-career	
Individual	Time/Funding	Strategic use of time and funding for collaborations	Providing time for junior scientists	*It’s not so much that I need more time, I need to be better able to use the time I have. To be more efficient by using resources that I know exist but haven't connected to.*
	Resources	Just-in-time materials		*Help scholars know what they don't know when they need to know it*
	Identity	Effects of identity on career progress and promotion and tenure	Awareness of unique career opportunity	*With so much opportunity, it’s easy to get whiplash and there is no one research career, it is so varied. When my path is off the classic recipe, it makes me feel like an imposter. With CTR, I don't fit squarely in a single discipline, but can fit in almost any. I use different words, but the ideas fit the same*
Proximal	Mentorship	Deepening connections to senior scholars	Mentor-precision recommendations	*I need help getting a lay of the land, using things like individual development plans. Maybe there are other opportunities like externships or building relationships outside academic settings that I do not know*
	Relationships	Building mutually beneficial connections with peers and scholars	Reduce research redundancy through connections; rolodex of individuals; events to facilitate connection	*ICTR can identify what [trainee] objectives are, then create win-win interactions between different players, up and down this chain of interacting decision-making agents who … agree to work together and find it mutually beneficial to do what it takes to get over hurdles that get in the way of translation.*
	Team Science	Building a successful team earlier	Interdisciplinary; cross-cross-spectrum team science building	*The job is simply too big for any individual to take on by themselves. It takes a lot of different players to get aligned and set up with proper incentives in place, like promotion and tenure recognition, to collaborate to take good ideas and advance them through each stage.*
Institutional	Build Network/Hub Capacity for CTR and clinical trials	Build CTR incentives and reduce hurdles to clinical trials	Build CTR scaffolding for scientists; reduce systemic barriers, especially in IRB and EHR access	*Need better answers for how we build a system as an institution that really supports translational scientists. ICTR could provide the kind of big picture system design thinking… With a huge, complex, multi-player coordination kind of process, things can fall apart at any level. ICTR could be the coordinator of the pieces of CTR.*
	Build more encouragement of CTR	Increase acceptance of CTR for promotion and tenure		*One problem is working through regulatory research. Some colleagues doing lab research won't do human research because the hurdles are high and it’s time consuming. We do well training people what regulations are, but logistics of getting things done can be daunting*
Inter-institutional	Accessing Communities	Align community interaction with early-career goals; deepen connections to communities; Building trust	Build resources to locate and interact with more diverse communities	*How do we organize bringing together all of the players scattered all over? We need to roll-on systems that connect people and places, with systems to support that work. And account for the complexity of the undertaking earlier in the research process*

CTR = Clinical and Translational Research; ICTR = Institute for Clinical and Translational Research; IRB = Institutional Review Board; EHR = Electronic Health Record.

To identify specific elements of discourse underlying the challenges and opportunities of CTR, we prepared a set of qualitative codes (Table 1) representing explicit themes. Qualitative codes were assigned to the transcripts, showing if a code was present (1) or not present (0) within each line of transcript. Coders isolated, and later agreed upon, lines of text that represented quotes related to specific themes. Those themes were then mapped to the layers of the ecological model, including individual, proximal, institutional, and inter-institutional layers (Table 1). As we analyzed the data, we began to observe distinct sets of responses from early career as compared to senior-career CTR scientists. To that end, we categorized the interviewees into two groups: (1) early-career experts who ranged from pre-tenure to up to 3 years tenured (*n* = 9), and (2) senior-career experts who were late-stage experts with more than 5 years post tenure (*n* = 6). Subsequent analyses allowed us to better understand the unique needs of scientists based on experience and identity.

To compare those two groups of scientists, we used ENA (described elsewhere and available online [25,26]) which identifies patterns in themes across respondents and models those patterns. ENA is a modeling technique for qualitative discourse data that captures specific thematic elements. The analysis quantifies times when themes (here represented as codes) co-occur within defined segments of data. ENA visualizes that communication, weighted, in a multidimensional model. ENA allows for both counts of word utterances and patterns amongst those utterances, thus connections between thematic content can be found. Thus, results of ENA more closely mirror the interrelatedness of content from real-life authentic conversations than what can be achieved by counting techniques alone [27]. Here, the data were analyzed using moving windows [28], which means that the co-occurrence of codes was sought within four-paragraph sets. Themes were considered co-occurring if they were found within those 4-paragraph “windows.” Resulting co-occurrences, here the differences in needs for early-career and senior-career scientists, could be tested across diverse conditions or groups [25,27].

**Fig. 3.**
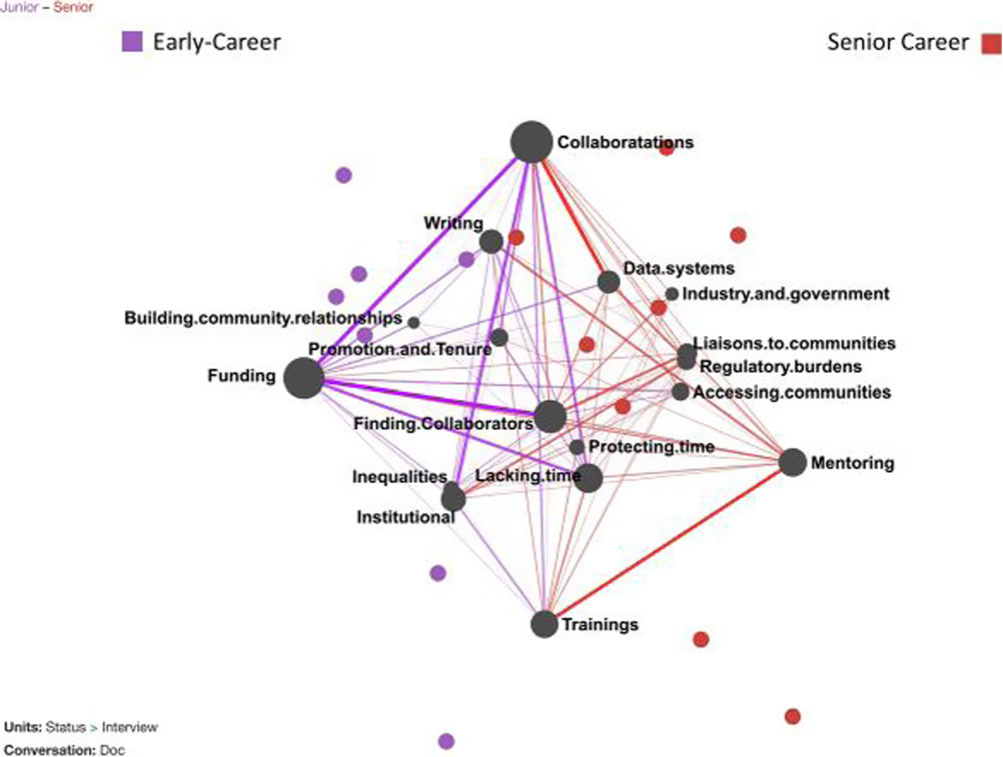
Mapping of epistemic analytics linguistic observations* in interviews between early-career (purple) and senior-career (red) clinical and translational research scientists. *Thicker lines represent more frequent connections and wider circles represent codes that are more frequently connected.

Using the moving window method, ENA visualized a network model (Fig. 3) for each segment showing how thematic codes connected to other codes within the temporal context [29,30]—defined here as four paragraphs preceding each utterance. ENA resulted in network models for each interviewee, accompanied by a dimension reduction algorithm called a means rotation [31]. That algorithm projected the distance between utterances on hypothetical planes that (1) maximizes the difference between codes across groups – here, early-career and senior-career scientists – and (2) models singular value decomposition to isolate unique codes. Subsequently, authors used ENA to create two representations, one for early-career and one for senior-career scientists (Fig. 3), where nodes corresponded to codes and the weight of edges (e.g., connecting lines) reflected the relative frequency of connections between two codes, and a plotted point. Thus, the ENA quantified and visualized the structure of connections among themes and compared differences across experience levels, making it possible to characterize CTR codes within and across those levels. To test whether the differences between career stages were statistically significant, the ENA was tested using Mann–Whitney U tests to compare the mean ENA scores on each theme between groups, isolating statistical differences between groups (Table 2). After analyzing the data using ENA, the team of coders and ENA expert revisited the qualitative data to further investigate key connections between themes, ecological layers, and career stage.

**Table 2.** Frequency and average mentions of themes across early-career and senior-career scientist interviews using Epistemic Network Analysis

Level	Themes	Total theme count		Average mention	
		Early-career	Senior-career	Early-career	Senior-career
Individual	Writing	21	23	3.50	2.56
	Funding	49	40	8.17	4.44*
	Promotion and Tenure	11	5	1.83	0.56*
	Lacking time	25	22	4.17	2.44*
	Protecting time	5	22	0.83	2.44*
Proximal	Trainings	29	49	4.83	5.44
	Mentoring	10	25	1.67	2.78*
	Collaborations	66	77	11.00	8.56*
	Finding Collaborators	37	49	6.17	5.44
	Industry and government	1	10	0.17	1.11*
	Accessing communities	12	16	2.00	1.78
Institutional	Regulatory burdens	4	29	0.67	3.22*
	Data systems	14	37	2.33	4.11*
	Inequalities	23	25	3.83	2.78
Inter-Institutional	Liaisons to communities	3	11	0.50	1.22*
	Building community relationships	5	4	0.83	0.44

* *p* < 0.0001 differences between early-career and senior-career Clinical and Translational Research scientists in frequency of mentions.

### Quantitative Survey

Upon completion of the qualitative interviews, the Oversight Committee developed a quantitative survey – distributed to the current CTR trainees – to assess the prevalence of needs, gaps, and barriers that resulted from qualitative interviews. The focus of each survey item mirrored the qualitative questions. That yielded a brief four-item web-based quantitative survey (available in the Supplemental Digital Appendix 1) of active ICTR trainees across multiple training programs. Questions inquired about individual supports, university-wide characteristics, connections to individuals or groups, and training, webinar, and educational materials of interest. Response sets were created by classifying the primary responses from qualitative interviewees into distinct categories and allowing for an open-ended “other” response. The survey was assessed for content-related validity, including face validity and item validity, by the Oversight Committee. Criterion-related validity, such as predictive validity, and construct-related validity, such as discriminant validity or generalizability, were outside of the scope of the needs assessment.

Interviewees from the qualitative interviews were precluded from participating in the quantitative survey. REDCap surveys were sent to all existing trainees, with two follow-up requests for completion. A 50% response rate yielded 68 completed surveys from active CTR trainees. Considering the intentions of this needs assessment, we deemed this an adequate response rate. The impact of nonresponse depends upon multiple factors [32], including (1) the relationship between the outcome of interest and the decision to participate in the survey, (2) the relative importance of generalizing to a population-based estimate, and (3) the necessity of calculating an absolute estimate. As our needs assessment encompassed a relatively small distinct group and was exploratory in nature, we believed a higher response would not add to variability in responses. Our response rate was consistent with expectations for response in higher education surveys and beyond the recommended minimum of 50 respondents identified in previous studies [33].

The identities of survey respondents mirrored the diversity of identities in our training class. The survey yielded 57% female- and 43% male-identified respondents, from a small range of diverse ethnic backgrounds (8% African American or Black; 15% Asian or Asian American; 3% Chicano, Latino or Hispanic; 9% multiethnic; 64% White). More than half of survey respondents had a current Assistant, Associate, or Full Professor title. Approximately 30% were graduate students and 20% also held Scientist or Researcher titles. Differences between groups on survey items, based on gender, ethnicity, or under-represented scientist, were calculated using non-parametric Mann–Whitney U tests due to small sample sizes, with corrections for the number of tests (Table 3).

**Table 3.** Rankings of types of support listed as “somewhat” or “meaningfully” advancing or benefiting Clinical and Translational Research (CTR) organized by type of system support, with differences by group representation

Individual supports	University or institutional support
Pilot funding Statistical support Protected time Grant writing Proposal writing^a^ Research coaching^b^ Career coaching^b^ Inclusive excellence^c^	Participant recruitment Relationships with communities Improved awareness of CTR Facilitate clinical trials Increased promotion and tenure incentives for CTR^a,c,d^

(*n* = 70)

a Non-White participants reported significantly more benefit from proposal writing support (*p* < .001).

b Women expressed more benefit from career and research coaching (*p* < .001).

c Historically under-represented (HU) participants reported more benefits from institutes that prioritize inclusive excellence (*p* < .001).

d Non-white and HU participants perceived more benefit from promotion and tenure incentives (*p* < .01).

Implications of both qualitative and quantitative findings were generated by conversations between the needs assessment Oversight Committee members, ICTR leadership, and key personnel engaged in training CTR scholars (e.g., component Principal Investigators and faculty). We prioritized integrating the results of all three methods in understanding areas of need across the layers of the ecological model. The ENA validated the presence of themes by group that was found in the thematic analysis. We gave priority to those themes from the qualitative study that were also supported by the quantitative survey.

## Results

Participants across the career trajectory identified CTR scientist needs within multiple layers of the ecological system – individual, proximal, institutional, and inter-institutional. Themes related to needs, gaps, and barriers were identified at each level of the ecological model and interviewee career stage. Qualitative analyses, both thematic analysis (Table 1) and ENA (Table 2), supported ways that CTR researchers prioritized needs, gaps, and barriers based on career stage. Using ENA, networks of thematic codes were modeled for early-career and senior-career scientists by averaging the strength of the connection across all moving windows for each theme. The differences, and relative connections, between those networks were calculated and visualized (Fig. 2). Along the *X*-axis, a Mann–Whitney test showed that the discourse of early-career scientists (Mdn = −1.44, *N* = 6) was statistically significantly different from senior-career scientists (Mdn = 0.80, *N* = 9 *U* = 0, *p* < 0.0001, *r* = 1.00).

### At the Individual Level

In qualitative interviews, early-career scientists expressed a need for specified support services, including requests for precision or tailored guidance specifically regarding how to strategically use time and funding to achieve career goals which also meet criterion for promotion and tenure. Interviewees used words including “*just-in-time,” “packaged materials,”* and “*self-service.”* Overall, early-career professionals were more concerned about individual-level issues (e.g., writing, funding) than senior colleagues (*p* < 0.0001, Table 2). The ENA revealed that early-career scientists made more frequent connections between needs and barriers related to funding and how funds are used (*p* < 0.0001, Table 2). Many requested a need for better awareness of what ICTR provides, navigation through available services, and increased capacity to use ICTR services. Rather than requests for *more* time, the request was for supports that would maximize the capacity to *use* time. To maximize research productivity and best use time, scientists advocated for help navigating through the enormous number of available resources, including better advertising of and explicit direction towards ICTR services based on what that scientist needed in that moment. Examples included a research navigator, as exists at other CTSA hubs, and access to cross-trained support staff who could readily complete specific tasks without delays or turnover.

Early-career scholars described “*limits”* and “*less freedom”* within the academic context that prioritizes quantity of publications, particularly in high-impact journals. Interviewees who identified as being relatively more marginalized – based on a marginalized identity or representing a nontraditional discipline within a School or college – described greater levels of struggle with meeting career goals (Table 3). Interviewees who were pre-tenure – thus also early-career – identified decision-making based on promotion and tenure that was disconnected from areas of personal passion or societal impact (Table 1). Specific examples included pressure to research areas with high publication potential versus building deeper relationships with community collaborators. Senior scientists also described the dilemma faced by early-career scientists: CTR research invites intensive community collaboration, which can be at odds with department expectations for maximum productivity in clinical and research areas.

Survey findings that asked scientists to reveal the “most beneficial” supports for their work identified needs at the individual level including pilot funding, statistical support, protected time, grant writing support, and proposal support. Comparisons were made between groups, based on identity, correcting for sample size. Non-white participants, and those who identified as historically underrepresented in academic medicine, perceived more benefit from support for proposal writing and institutions that prioritize inclusive excellence (*p* < 0.001, Table 3) and more benefit from promotion and tenure incentives (*p* < 0.01, Table 3). Women perceived more benefits from research and career coaching (*p* < 0.001, Table 3), which is consistent with what we heard from interviewees who articulated needs for specific interventions based on a person’s identity and department affiliation.

### At the Proximal Level

ENA indicated that early-career interviewees were more concerned about the proximal issues of building community relationships and finding collaborators than were senior-career scientists (Table 2). Individuals from underrepresented groups expressed the importance of exposure to successful researchers who shared their identities. Early-career scientists identified needing deeper connections to collaborators and senior scientists (*p* < 0.0001, Table 2), earlier in training, with particular attention focused at creating *meaningful*, *purposeful*, *sustainable* connections to inter-disciplinary researchers across the translational stages. Early-career participants also identified a desire to build relationships with people across labs, clinics, and communities earlier in the process. In interviews, scientists identified struggling with building in-depth community relationships amid the many other clinical demands in academic medicine. Specific requests included a liaison program to connect researchers with collaborators and communities that advance team science. Participants identified that a few successful “match making” programs exist for research scientists, but more would be helpful. ENA revealed a difference in proximal concerns between early-career and senior-career scientists: while the early-career professionals were more interested in career-building proximal concerns, the senior scientists were more concerned about research trainings, mentoring, and access to industry and government (*p* < 0.0001, Table 2). In interviews, senior scientists identified that increased collaboration between individuals and departments would help to reduce research redundancy and under-enrolled research studies at the university.

### At the Institutional and Inter-Institutional Level

Senior scientists were more likely to identify system-level characteristics that would advance CTR. Interviewees used analogies that presented the CTSA as a *general contractor for CTR*, speaking of the position the CTSA can play in building necessary links between education, individuals, content, regulatory bodies, and health research opportunities. Senior-career scientists were more concerned with institutional and inter-institutional concerns such as data systems and creating community liaisons, with others identifying institutional costs associated with regulatory hurdles (*p* < 0.0001, Table 2). Requests were made for increased access to and utility of data and data systems, with a special focus on data usage and security policies. Scientists advocated for increasing promotion of CTR within academic medicine, earlier collaboration planning for later stages of translational research, increased capacity for successful clinical trials, and building access to trials that integrate across multiple disciplines.

Survey respondents identified a need for more community engagement and building a community where CTR is encouraged (Table 3). Women perceived more advancement from support for community engagement (*p* < .001). Non-white and HU participants perceived more benefit from P&T incentives (*p* < .001). Two university characteristics were rated as negatively impacting career success, including difficulties related to complex IRB policies and hardships accessing electronic health records.

## Discussion

The goal of this research was to better understand the needs of CTR scientists going into the future, with the aim to improve our CTSA education and training interventions. Interview and survey participants provided suggestions for intervention, both generalized and specific, related to improving individual training, deepening dyadic relationships, shifting beliefs about CTR, and building policies and procedures that facilitate CTR career growth. While this assessment was conducted for a single hub, based upon similarities in our findings and reports across multiple other studies, these results may be helpful for other CTSA Hubs. This study resulted in these primary findings: 1) scientists’ needs depend upon where a scientist is in their career trajectory and early-career scientists seek elevation of CTR in performance and tenure decision-making; 2) scientists want earlier, more specific, direction to available resources, 3) scientists need more active support for building teams – support for inter-disciplinary connections, community engagement, and team-based incentives, and 4) understanding needs is critical for the design of CTSA interventions.

Our findings validate the conclusion that specific supports are needed for early-career scholars, who would benefit from facilitated access to guidance navigating the research enterprise. While we expect all scientists benefit from having more time, early-career scientists indicated needing guidance for better using the time they have. How to approach this problem? As van Dijk and colleagues have identified [6], specific supports like a stronger awareness of CTR and more scholarly incentives for CTR are critically important. As Hsiao and colleagues [34] advocate, CTSA hubs can support trainees at different points along the career trajectory. Building a supportive environment, with all of the complexities of CTR, takes efficient, streamlined approaches to navigating available supports and services. It is critical to understand unique barriers experienced by those who have been more marginalized to level the playing field and build specific supports that embrace multiple perspectives and disciplines. Using culturally aware mentoring [35,36] is also important for those who identify as under-represented, who describe having more frustration or higher hurdles to overcome than others. Consistent with the aims of Team Science [8], specific supports should also include building strong inter-disciplinary, community-engaged [11], forward-looking relationships. Doing so may decrease attrition due to frustration that emerges with hurdles and disincentives. Individual scientists benefit when approaching research design, execution, and implementation in a team.

Of critical importance is ameliorating the logjam that can occur at the intersection of CTR and promotion and tenure policy. Quantitative findings support that there is a need for building deep, meaningful, connections to communities for the purpose of translational research, which is critical to advancing health equity and minimizing health disparities [37]. As CTSAs advance science careers in ways that align with tenure expectations, several CTR supports are required, including access to supportive resources, training, mentorship, tenure clock extensions, allowing for early community engagement, and building policies that support the time required to develop a niche in human subjects research [6].

While these interviews did not lead to comprehensive plans for how to implement training or policy improvements, the Organizing Committee and local ICTR experts designed a list of recommended interventions based on these findings (Table 4). Interventions should be across the translational spectrum and consider ways to intervene at all levels of the system. Both assessing and strengthening existing systems, as well as increasing advocacy and awareness of existing systems, are warranted. Senior scientists have the experience and power to support decision-making and efficiencies at each layer of the system. As an example, one early-career scientist described benefitting from a networking event designed by a senior-career scientist to introduce them to other scientists, a major grant of which emerged from that encounter. Our findings support an approach that invites and deploys critical individuals in strategic ways. By sharing best practices, and building cross-CTSA solutions, we maximize scientist outcomes and level the playing field for scientists from all backgrounds and disciplines.

**Table 4.** Interventions based upon systems level

Level	Intervention
Individual	* Facilitate scholarly identities with Individual Development Plans [39,40] * Personalized resiliency training related to scholar identity [38,41,42] * Ease navigation of CTR resources (e.g., single entry point, JIT, packaged materials) * Increase informatics/statistical and clinical trial training
Proximal	* Increase opportunities for interdisciplinary scholarly connection [43,44] * Build Communities of Practice for research collaborations [45,46] * Facilitate collaboration planning [47] * Cross-train research support staff * Build more welcoming, supportive communities for diverse scholars [48]
Institutional	* Increase institutional support for CTR [6] * Improve institutional support for clinical trials * Decrease regulatory burden and institutional barriers (e.g., SMART IRB, protocol development support) * Increase access to data/data systems * Facilitate relationship-building with special populations [37,49]
Inter-Institutional	* Build and facilitate access to communities [15,50] * Increase access to EHR data [51] * Build externships with communities and industry

*CTR = Clinical and Translational Research; EHR = Electronic Health Record; JIT = just in time; SMART IRB = Master Common Reciprocal Institutional Review Board.

### Limitations

A benefit of our approach was the ability to talk to an extremely diverse group of qualified, successful, CTR scientists. The collective wisdom of those scientists has tremendous value. That said, results speak to a small group of individuals engaging in research within a single Midwestern state at a single point in time. These data shed light on a few notable issues related to tailoring training based on the developmental needs of scientists, while advocating uniquely within institutional settings to create policies and procedures that support early-career scientists. Though this user needs assessment was conducted within the period of the global pandemic, primarily during phases of vaccine development, many CTR scientists were transitioning their expertise and resources to better understand the emerging pandemic and actively reflecting upon areas of need. Other needs assessments [38], also conducted during COVID-19, have reported similar differences in experience by gender and minority status. While the pandemic is likely to have colored scientist responses to our qualitative and quantitative questions, many participants indicated that they had additional time to reflect upon their needs and evaluate and assess new opportunities while remote working during the pandemic. Though participants mentioned COVID-19 during the interviews, it was not elevated to a unique code which suggests that perceptions of needs and resources in this assessment were not driven by COVID-19 conditions.

Bias, in the way of positive perceptions of ICTR, existed across research phases as many participants were funded or employed by, or in some other way affiliated professionally with ICTR. Persistent reviews like “ICTR does amazing work” and “ICTR has significantly supported my career to date” were anticipated and omitted as inconsistent with the goal of this assessment. The goal of analyzing these data was to identify areas for additional growth and support not currently accessible at ICTR.

## Conclusion

The success of the CTSA program to advance science, medical interventions, and the health of communities is well-documented. By sharing our results, which we observe to hold true at other CTSA hubs, we hope to advance our collective quality improvements. As a result of our learning, it is our intent to conduct a similar user needs assessment every 2 years throughout each renewal cycle. The learning helps us to effectively, ethically, and responsibly invest funds to improve the livelihoods of all scientists.

## Supplementary material

For supplementary material accompanying this paper visit https://doi.org/10.1017/cts.2023.6. click here to view supplementary material
