# Supplementary material for: Win–win interactions: Results and implications of a user needs assessment of clinical and translational scientists
Source: J Clin Transl Sci. 2023 Feb 7;7(1):e73. doi: 10.1017/cts.2023.6 (PMC10052438; doi:10.1017/cts.2023.6)
Supplement: Supplementary file 1 [file S2059866123000067sup001.docx]

**JCTS_RA_22_0163**

**Win-Win Interactions: Results and Implications of a User Needs Assessment of Clinical and Translational Scientists**

Supplemental Digital Appendix 1:

Survey Items:

1.) To what extent would the following individual supports benefit your career or your clinical and translational research (CTR)? Response set: Pilot project funding, Protected time, Statistical consultation, Grant management support, Proposal development support, Commercial development support, Career coaching, Research coaching, Deeper understanding of inclusive excellence

2.) To what extent do the following university-wide characteristics serve to advance *or* limit your clinical and translational research efforts? Response set: IRB procedures, EHR policies, EHR access, Scholarly incentives like tenure or promotion, Acceptance or promotion of CTR by others, Institutional support for clinical trials, Institutional support for community engagement, Institutional support for participant recruitment & retention

3.) How likely would you be to seek out a training, webinar, or educational materials on these topics to improve your work? Response set: assessing research/clinical impact, making research more culturally inclusive, dissemination & implementation, medical or technological innovations, data management, clinical trial implementation, team science, integrating work and home demands, advancing research along the translational science spectrum, evaluation tools & techniques
